# Management of Aggressive Non-Hodgkin Lymphomas in the Pediatric, Adolescent, and Young Adult Population: An Adult vs. Pediatric Perspective

**DOI:** 10.3390/cancers14122912

**Published:** 2022-06-13

**Authors:** Irtiza N. Sheikh, Amr Elgehiny, Dristhi Ragoonanan, Kris M. Mahadeo, Yago Nieto, Sajad Khazal

**Affiliations:** 1 Department of Pediatrics, Pediatric Hematology Oncology, The University of Texas MD Anderson Cancer Center, Houston, TX 77030, USA; isheikh1@mdanderson.org; 2 Department of Pediatrics, McGovern Medical School, The University of Texas at Houston Health Science Center, Houston, TX 77030, USA; amr.i.elgehiny@uth.tmc.edu; 3 Department of Pediatrics, CARTOX Program, Pediatric Stem Cell Transplantation and Cellular Therapy, The University of Texas MD Anderson Cancer Center, Houston, TX 77030, USA; dragoonanan@mdanderson.org (D.R.); kmmahadeo@mdanderson.org (K.M.M.); 4 Department of Stem Cell Transplantation, The University of Texas MD Anderson Cancer Center, Houston, TX 77030, USA; ynieto@mdanderson.org

**Keywords:** non-Hodgkin lymphoma, pediatric, adolescent, immunotherapy, CAR T-cell therapy, hematopoietic stem cell transplantation, lymphoma, diffuse large B-cell lymphoma

## Abstract

**Simple Summary:**

This review details the diagnosis and treatment of primary non-Hodgkin lymphoma (NHL) in the pediatric and adolescent population. We also describe treatment modalities such as hematopoietic stem cell transplantation for relapsed or refractory NHL in patients that fail or do not respond to the initial therapy. We then detail the current advancements in treatment for patients that fail initial therapy such as CAR T-cell therapy, the use of immunotherapy that target surface makers on malignant cells and highlight areas where further research is needed. The purpose of our review is to inform the pediatric oncology community in regard to the various types of NHLs and emphasize areas where the science is evolving in the treatment of primary, relapsed or refractory disease.

**Abstract:**

Non-Hodgkin lymphoma (NHL) is a broad entity which comprises a number of different types of lymphomatous malignancies. In the pediatric and adolescent population, the type and prognosis of NHL varies by age and gender. In comparison to adults, pediatric and adolescent patients generally have better outcomes following treatment for primary NHL. However, relapsed/refractory (R/R) disease is associated with poorer outcomes in many types of NHL such as diffuse large B cell lymphoma and Burkitt lymphoma. Newer therapies have been approved in the use of primary NHL in the pediatric and adolescent population such as Rituximab and other therapies such as chimeric antigen receptor T-cell (CAR T-cell) therapy are under investigation for the treatment of R/R NHL. In this review, we feature the characteristics, diagnosis, and treatments of the most common NHLs in the pediatric and adolescent population and also highlight the differences that exist between pediatric and adult disease. We then detail the areas of treatment advances such as immunotherapy with CAR T-cells, brentuximab vedotin, and blinatumomab as well as cell cycle inhibitors and describe areas where further research is needed. The aim of this review is to juxtapose established research regarding pediatric and adolescent NHL with recent advancements as well as highlight treatment gaps where more investigation is needed.

## 1. Introduction

Non-Hodgkin lymphoma (NHL) encompasses a complex set of lymphomatous tumors. More than 50 different types of NHL have been classified by the World Health Organization [1,2]. The prevalence, outcomes, and treatments of NHL differ depending on its type [3]. Moreover, it is important to distinguish between pediatric and adolescent NHLs and those seen in adults because diagnostic markers and outcomes can differ widely between the two populations. While children with most common types of NHL have excellent event-free survival (EFS) and overall survival (OS) rates, those with relapsed or refractory (R/R) NHL have poor outcomes despite advances in salvage chemotherapy and hematopoietic stem cell transplantation (HSCT). Moreover, current treatment regimens for NHL are associated with adverse effects such as mucositis, decreased appetite, and risks to fertility [4]. Recent advances in the treatment of NHL, such as the combination of rituximab, an anti-CD20 monoclonal antibody, with multiagent chemotherapy regimens or the use of brentuximab, an anti-CD30 antibody-drug conjugate, have improved EFS and OS rates in pediatric and adolescent patients with primary NHL [5]. In this literature review, we discuss the molecular and clinical characteristics of pediatric and adolescent NHL and compare them to those seen in adults with NHL. We then discuss the conventional treatment of primary NHL as well as that of R/R disease. We end the review by describing recent advances in the treatment of NHL, including immunotherapies such as chimeric antigen receptor (CAR) T-cell therapy and cell cycle checkpoint inhibition, and highlighting areas that require further research, for instance whether targeted drugs can improve survival rates of patients with R/R disease.

## 2. Epidemiology of Non-Hodgkin Lymphoma in Pediatric and Adolescent Patients

The most common types of NHL observed in the pediatric population include Burkitt lymphoma, diffuse large B-cell lymphoma (DLBCL), lymphoblastic lymphoma (both B and T cell origin) while less common types such as anaplastic large-cell lymphoma (ALCL), primary mediastinal large B-cell lymphoma (PMLBCL), and follicular lymphoma are also seen [6]. The type and prevalence of NHL in children and adolescent varies by age, and outcomes depend on both age and sex [7]. Two of the most common types of NHL in the pediatric population are Burkitt lymphoma, which arise from B lymphocytes, and lymphoblastic lymphoma, of which nearly 80% arise from T cells, with the rest originating from B cells, especially in patients from 1 to 9 years old. As children age, the incidence of Burkitt lymphoma decreases, whereas the incidence of DLBCL markedly increases, especially in 15–19-year-olds [6]. In those less than 20 years of age, NHL is relatively uncommon, comprising only 7% of all cancers. In children younger than 5, the incidence is about 6 per million and in adolescents about 15 per million [8].

Moreover, outcomes are markedly better in pediatric and adolescent patients with NHL than in adults [6]. Data from the National Cancer Institute’s Surveillance, Epidemiology and End Results (SEER) program show that over a 24-year period, the prevalence of NHL amongst all cancers has increased by more than 30%; however, over a 10-year period, the survival rate of NHL has improved by nearly 20% with all subgroups including infants, pediatrics, and adolescents having a 10-year survival rate of more than 60%. Patients between the ages of 1 and 4 years account for the best improvement, with a 10-year survival rate of 78% [9]. A review of data from 13 SEER registries that included patients younger than 30 found that males were diagnosed with NHL more often than females, with prevalence rates of 60% and 30% of NHL cases, respectively [10]. However, a study of pediatric patients with NHL found that adolescents (15–18 years old) and girls were typically diagnosed with more advanced NHL (>stage II) and that girls had lower 5-year EFS rates than did boys (70% vs. 83%, respectively) [11].

## 3. Pathophysiology, Clinical Outcomes, and Clinical Characteristics of Pediatric and Adolescent Non-Hodgkin Lymphoma

### 3.1. Burkitt Lymphoma

Burkitt lymphoma involves intermediate-sized B cells expressing the surface markers CD19, CD20, and immunoglobulin kappa or gamma light chains. These cells can infiltrate nodal or extra-nodal tissue, and their rate of cell division is considered to be the highest of any human tumor. Thus, Burkitt lymphoma is a highly aggressive type of NHL [12,13]. The chromosomal translocation characteristic of Burkitt lymphoma involves the distal region of the long arm of chromosome 14 and the long arm of chromosome 8 (t(8;14)), which leads to translocation of the oncogene *MYC* to one of the loci encoding for the immunoglobulin heavy chain [14]. This leads to activation of *MYC* and the malignant transformation of B-type lymphoma cells [15]. Burkitt lymphoma is classified into three types: endemic, sporadic, and immunodeficiency-related (e.g., those associated with HIV infection) [16,17]. Burkitt lymphoma was one of the first malignancies to be associated with a virus when Epstein et al. [18,19] isolated viral particles, later named the Epstein Barr virus (EBV), from lymphomatous cells. The endemic type of Burkitt lymphoma is closely associated with EBV and malaria infections and is especially prevalent in equatorial regions of Africa, South America, and Oceania, such as Papua New Guinea [20,21,22]. The endemic form most commonly affects the jaw, whereas the sporadic form is most often seen in the abdomen followed by the head and neck region (not including the jaw), the bone marrow, and the central nervous system (CNS) [17,23]. The general presentation of patients with BL includes a rapidly enlarging mass, lab values indicating spontaneous tumor lysis, such as increased uric acid or lactate dehydrogenase, and in those with abdominal disease, abdominal distention and nausea/vomiting [17]. Moreover, in pediatric patients outside the common age in which intussusception may be seen, intussusception may be the presenting symptom, requiring surgical excision and the disease can be associated with early-stage disease with favorable response following surgical resection and chemotherapy [24,25].

### 3.2. Diffuse Large B-Cell Lymphoma

Although DLBCL is the most common NHL in adults, it accounts for about 10–20% of NHLs in the pediatric and adolescent population. The clinical presentation is most consistent with diffuse lymphadenopathy in areas like the head and neck and inguinal regions, hepato-splenomegaly, and B-symptoms including fever, weight loss, and night sweats [26]. Extra-nodal involvement may also include the abdomen followed by the skin, with infiltration of other organs also a possibility. Clinical emergencies may include superior vena cava (SVC) syndrome and compression of the large airways, leading to respiratory distress [26].

Like Burkitt lymphoma, pediatric DLBCL is an aggressive malignancy [23]. However, the EFS rate of pediatric and adolescent patients with DLBCL is nearly 90%, compared in comparison to about 50% in adults, including those treated with rituximab [27,28,29]. In adult DLBCL, germinal center DLBCL is the most common subtype, comprising about 40–50% of cases, and carries a better prognosis than the activated B-cell-like subtype (5-year OS rates of 59% vs. 30%, respectively). In contrast, in the pediatric and adolescent population (from 0 to 19 years of age), more than 70% of patients with NHL have germinal center-type DLBCL. This subtype of DLBCL is characterized by CD10 and BCL6 expression. Moreover, unlike the adult version of DLBCL where the overexpression of BCL2 protein due to t(14;18) occurs in 20–30% of cases, the incidence of the translocation is believed to be much lower in pediatric patients [30,31,32,33].

### 3.3. Primary Mediastinal Large B-Cell Lymphoma

Primary MLBCL (PMLBCL) was once considered a type of DLBCL but is now considered a distinct entity in the NHL class and makes up nearly 2% of NHLs in patients younger than 18 [1,34]. The clinical presentation is most likely to manifest with involvement of the upper mediastinum, leading to involvement of the chest wall, heart, lungs, and pleura with associated pleura/pericardial effusions [35]. Similar to DLBCL, PMLBCL may also be associated with SVC syndrome and respiratory distress due to airway compression [36].

PMLBCL been found to be associated with gain-of-function mutations involving chromosome 9, where the genes *JAK2*, *PDL1*, and *PDL2* are located, as well as mutations affecting the STAT pathway that lead to constitutive *STAT6* activation. Activation of *STAT6* can lead to evasion of apoptosis and promotion of cell proliferation [23,37,38]. The pathologic characteristics of PMLBCL tumors can vary; some cells show pale cytoplasm, moderately sized nuclei, and sclerosis that overlaps with features of classical Hodgkin lymphoma. Cell surface markers include CD19, CD20, CD30, and CD79a [37,39]. While primary involvement of the mediastinum, including the lungs, pleura, and pericardium, is common, and spread to organs such as the kidneys or liver may occur, CNS and bone marrow involvement is generally rare [40,41]. However, advancements in genetic testing through methods such as fluorescence in situ hybridization have facilitated detection of *CIITA* and *PDL1/PDL2* genes in cases of extra-mediastinal PMLBCL that were otherwise classified as DLBCL, indicating that tumor genetic testing may help recognize other cases of extra-mediastinal PMLBCL erroneously classified as other NHLs [42]. PMLBCL generally carries a favorable prognosis, as shown by a Children’s Cancer Group (CCG) study in which 100% of patients demonstrated a complete response (CR), despite exposure to three different regimens, with EFS and OS rates of 75% and 85%, respectively. However, these rates are worse than those in localized large-cell lymphoma, which had EFS and OS rates of 92% and 97%, respectively [43].

### 3.4. Lymphoblastic Lymphoma

Lymphoblastic lymphoma is the second most common NHL in children, accounting for nearly a quarter to a third of all pediatric NHL cases with a bimodal age of distribution, seen at a median age of 1–4 years old and 15–19 years old [44]. Its EFS and OS rates are both greater than 80% [45]. Lymphoblastic lymphoma most commonly arises from T lymphoblasts, but 20% of cases arise from B-cell lymphoblasts. Lymphoblastic lymphoma affects boys twice as often as girls [46]. Nearly a quarter of T-cell lymphoblastic lymphoma cases affect the mediastinum, and the disease can also be found to affect the bone marrow (BM); by comparison the disease is distinguished from T-cell leukemia considering that T-cell lymphoblastic lymphoma does not have more than 25% of BM involvement [47]. Moreover, recent genetic testing has revealed that mutation of *NOTCH1*/*FBXW1* genes may reveal a disease that portends a good prognosis and gene mutations may allow risk stratification to occur as well as help elucidate T-cell lymphoblastic lymphoma as more distinct than T-cell leukemia [48,49]. More than 90% of T-cell lymphoblastic lymphomas express terminal deoxynucleotidyl transferase, CD2, CD3, and CD10, and about 20% express CD34 [47]. In a study conducted by the COG, CD33 expression and bone marrow involvement did not appear to significantly affect EFS, which was 86%; however, increasing bone marrow involvement did affect the 4-year OS rate, which was 88% in patients without bone marrow involvement but about 75% in those with bone marrow involvement [47].

### 3.5. Pediatric Follicular Lymphoma

Pediatric follicular lymphoma (PFL) is a rare NHL that is characterized by local lymphadenopathy and stage I/II disease. It can generally be cured by conservative management, such as surgical resection, without systemic chemotherapy or radiation. Unlike its adult counterpart, PFL is not known to recur after therapy [50,51,52]. In adults, follicular lymphoma makes up more than 20% of NHL cases, making it the second most common type of NHL, but in children and adolescents, PFL accounts for fewer than 2% of NHLs [52]. Owing to the rarity of PFL, the literature lacks detailed treatment guidelines or histopathologic characteristics [51]. Despite the lack of standard treatment, the EFS rate for patients with PFL is about 90% [53]. Unlike adult follicular lymphoma, in which 75–80% of patients harbor t(14;18), PFL is characterized by a lack of *BCL2* rearrangement or over-expression of the bcl-2 protein [53,54].

### 3.6. Anaplastic Large-Cell Lymphoma

Anaplastic large cell lymphoma (ALCL) is a T-cell lymphoma that is rare in children and adolescents, with a median age of presentation of 16 years old, making up about 10% of NHLs in this population [44]. The disease generally presents in advanced stages (III-IV) with abdominal and mediastinal lymph node involvement as well as B-symptoms [55]. While BM disease is not common (<15% of cases), extra nodal spread to organs such as the skin, liver, and lung may be seen [56]. ALCL is characterized by large cells that express CD30; studies show that about 80% of cases harbor t(2;5), which encodes an NPM-ALK fusion protein. In murine hematopoietic stem cells, the introduction of this protein leads to malignant transformation [57,58]. In pediatric patients with ALCL, the overexpression of certain cytokines such as IL-6 and IL-2R also indicates that these patients have a distinct cytokine profile, although it is unclear if these makers have prognostic significance or signify a higher risk of developing ALCL [59]. There are no standard treatment guidelines [11,58]. However, ALK-positive disease appears to confer a better response to chemotherapy compared to ALK-negative ALCL [60]. In the pediatric population, patients with ALK-positive disease that involves the bone marrow have been found to have a 5-year PFS rate of 41% while those without bone marrow involvement have a 5-year PFS rate of 100%, indicating that bone marrow evaluation during the time of diagnosis may identify those that at risk of R/R disease [61].

The molecular characteristics and genetic abnormalities of the various pediatric and adolescent NHL types are described in Table 1.

## 4. Conventional Treatment of NHL in Children and Adolescents

The treatment of pediatric and adolescent NHL mainly consists of multidrug chemotherapy; radiation is not generally used in newly diagnosed or low-grade cases [3]. Moreover, unlike adult NHLs, certain pediatric NHLs, such as Burkitt lymphoma and DLBCL, share the same treatment backbone [7]. The treatment backbone of each pediatric and adolescent non-Hodgkin lymphoma is summarized in Table 1.

### 4.1. Treatment of Burkitt Lymphoma and DLBCL

Prior to the addition of rituximab to the systemic chemotherapy regimen for Burkitt lymphoma, treatment generally consisted of three phases: reduction, induction, and consolidation. The regimen is based on the FAB LMB 96 international study, which used a lower dose of cyclophosphamide than did prior trials [76]. The treatment regimens are further detailed in Table 1. Patte et al. [76] demonstrated that in patients with intermediate-risk Burkitt lymphoma or DLBCL which they defined as stage I/II that was not resected or stage III/IV without CNS involvement, the 4-year EFS rate was 90% and the OS rate was nearly 93%. That study indicated that a subgroup of pediatric and adolescent patients with intermediate-risk Burkitt lymphoma or DLBCL can be treated with 4 courses of a multidrug regimen, allowing the use of reduced doses of cyclophosphamide (3.3 g/m^2^) and doxorubicin (120 mg/m^2^) [76]. In those with high risk DLBCL or Burkitt lymphoma, defined by stage III disease with elevated lactate dehydrogenase (LDH) levels or stage IV disease, the risk of CNS disease is elevated along with the risk of relapse. However, we discuss below a phase II trial that shows the addition of rituximab to the LMB backbone for the treatment of upfront, high-grade, high-risk DLBCL and BL has been found to be associated with improved EFS rates compared to a group receiving chemotherapy only [77]. We also discuss improvements in prophylactic management of CNS disease associated with high-risk disease.

### 4.2. Treatment of PMLBCL

While there is no standard treatment for PMLBCL, the most common regimen used in the United States is DA-EPOCH-R (etoposide, doxorubicin, cyclophosphamide, and vincristine with rituximab) [41]. Moreover, whereas the adult regimen may include consolidative radiation because PMLBCL is radiosensitive, radiation therapy is generally not considered a part of upfront treatment in pediatric patients. Indeed, large studies of pediatric patients treated with radiation did not clearly establish a response to or a role for radiation [41,78]. In a 2019 study by Giulino-Roth et al. [78] of adult and pediatric patients with PMLBCL, patients who received DA-EPOCH-R had a 3-year EFS rate of nearly 86% and an OS rate of 95%; however, more children than adults developed thrombosis (46% vs. 23%, respectively). In contrast, a more recent phase 2 trial by Burke et al. [79] involving only pediatric (<18 years old) patients with PMLBCL demonstrated that treatment with DA-EPOCH-R did not confer better EFS or OS (70% and 85%, respectively) compared to control treatment which had similar rates. This international study also compared its results with historical survival rates from the FAB/LMB 96 study. In that study, patients with stage III MLBCL had 5-year EFS and OS rates of 66% and 73%, respectively, with a regimen consisting of pre-induction low-dose cyclophosphamide, vincristine, and prednisone followed by an induction course of COPADM (cyclophosphamide, prednisone, doxorubicin, and methotrexate) and a consolidation course of CYM (cytarabine and high-dose methotrexate) [34]. In the Berlin–Frankfurt–Munster (BFM) group study, pediatric patients received a pretreatment course of steroids and cyclophosphamide, followed by a regimen consisting of steroids, high-dose methotrexate, cytarabine, etoposide, doxorubicin, and cyclophosphamide or ifosfamide, with dose intensity based on tumor size and staging based on serum lactate dehydrogenase levels. The study found a 5-year EFS rate of 70%. Lactate dehydrogenase levels were found to have prognostic significance; patients with levels greater than or equal to 500 U/L had a higher risk of treatment failure, with an EFS rate of 40% [80]. Considering that multiple trials have demonstrated no significant differences in EFS and/or OS among regimens, there is still a need to find the optimal treatment that can produce EFS and OS rates comparable to those seen in other NHLs.

### 4.3. Treatment of ALCL

While there are no standard treatment guidelines for ALCL, these tumors are sensitive to a variety of chemotherapeutic regimens which are detailed in Table 1 [81]. For instance, in a study of patients enrolled in the NHL-BFM group trials, pediatric patients with ALK-positive ALCL had a 9-year EFS rate of 83% after receiving a short-term (5–7 month) chemotherapy regimen [66]. The French Society of Pediatric Oncology also conducted a study in pediatric ALCL in which patients received 2 courses of COPADM followed by a maintenance phase. This study found 3-year OS and EFS rates of 83% and 66%, respectively [82]. A COG study (ANHL0131) demonstrated that substituting vinblastine for vincristine in the standard consolidation regimen of doxorubicin, prednisone, and vincristine did not significantly affect EFS or OS rates. Patients who received vinblastine had EFS and OS rates of 79% and 87%, respectively, compared to the standard-therapy group’s rates of 80% and 91%, respectively [83].

### 4.4. Treatment of Lymphoblastic Lymphoma

The NHL-BFM 90 study demonstrated that patients with T-cell lymphoblastic lymphoma who are treated with a chemotherapy regimen similar to that used to treat T-cell acute lymphoblastic leukemia (T-ALL)—anthracycline and cyclophosphamide with no local radiation—had an excellent 5-year EFS rate of 90% [84,85]. One of the largest trials involving pediatric and adolescent patients with T-cell lymphoblastic lymphoma, COG AALL0434, demonstrated 4-year EFS and OS rates of 87% and 90%, respectively, in patients who received an augmented BFM regimen as detailed in Table 1 [69,70,71]. As further described below, whether introducing nelarabine to the chemotherapy backbone improves survival in T-cell lymphoblastic lymphoma has not yet been established, although in patients with T-ALL, nelarabine reduces the rate of CNS relapse. It has been found to be tolerable in patients with T-cell lymphoblastic lymphoma; neurological adverse events were the most common issue [69,85].

### 4.5. Treatment of PFL

As mentioned previously, PFL lacks a standard treatment regimen; however, good responses to treatment regimens as detailed in Table 1 [52]. Another study showed that treatment with R-CHOP was also effective and led to remission in 83% of patients aged 0–18 years over a median follow-up time of 31 months [86].

### 4.6. Treatment of R/R NHL

Although primary Burkitt lymphoma and DLBCL have 5-year EFS rates of nearly 90% in the pediatric population, patients with R/R NHL have substantially worse outcomes, with a cure rate of about 30% [87,88,89]. Among pediatric patients with R/R NHLs, those with PMLBCL have the best chance of survival; a study by Burkhardt et al. [89] evaluated over 600 patients and found 8-year survival rates of 57% for PMLBCL, 28% for Burkitt lymphoma, and 50% for DLBCL. In the same study, patients with DLBCL who did not undergo HSCT did not survive at 8 years. In the French LMB study of 773 pediatric patients, 4% of patients experienced relapse, mostly patients with Burkitt lymphoma [90]. In this study, the responses to first-line salvage chemotherapy with R-CYVE (rituximab, cytarabine, etoposide, and dexamethasone) and R-ICE (rituximab, ifosfamide, cyclophosphamide, and etoposide) were compared. The CR rate in patients receiving R-CYVE was 56%, compared to 43% in patients who received R-ICE [90]. Moreover, 85% of patients in the LMB study received rituximab alongside multiagent chemotherapy, but only about 30% of these patients survived, raising the question of the efficacy of rituximab. Overall, the R-ICE regimen appears to be the preferred salvage chemotherapy regimen, especially as studies have shown overall response rates of 60–70% [91,92].

Both allogeneic and autologous HSCT are options for pediatric patients with R/R NHL and appear to have similar outcomes [87]. A study by Rigaud et al. [90] detected no difference in 5-year survival rates between patients who underwent allogeneic or autologous transplants (50% and 54%, respectively). In a retrospective registry analysis by Gross et al. [93] of 182 pediatric patients with R/R NHL who underwent allogeneic or autologous HSCT, 5-year EFS rates were similar within disease types. In patients who received allogeneic or autologous HSCT for DLBCL, EFS rates were 50% and 52%, respectively. For patients with R/R Burkitt lymphoma, the EFS rates were 31% for allogeneic HSCT and 27% for autologous HSCT. Patients with R/R ALCL, too, had similar EFS rates after allogeneic or autologous HSCT (46% and 35%, respectively). In contrast, patients with lymphoblastic lymphoma had better outcomes after allogeneic HSCT, with a 5-year EFS rate of 40%, compared to 4% in patients who received autologous transplants. Outside of the results in patients with lymphoblastic lymphoma, the results are similar to those of a 10-year study by Giulino-Roth et al. [94] which found no difference in disease-free survival (DFS) in patients who underwent autologous or allogeneic HSCT, with DFS rates of 53% in both patient cohorts. However, outcomes were markedly different among disease types: patients with ALCL had the best outcomes, with 77% DFS and 83% OS, while the five included patients with Burkitt lymphoma all died 1–3 months following HSCT. Taken together, these studies demonstrate that HSCT, regardless of transplant source, represents a chance for cure with patients with R/R ALCL and DLBCL (OS rates of about 70% and 60%, respectively) but less so for patients with Burkitt lymphoma (OS of about 30%) [89,95,96,97]. In contrast, in pediatric and adolescent patients that relapse or fail to respond following first-line chemotherapy and do not progress to HSCT, overall survival is significantly lower at less than 25% underscoring the importance of HSCT to provide greater chance of survival [98].

Although autologous and allogeneic HSCT have similar EFS and OS rates, some researchers posit that allogeneic transplantation may induce a graft-versus-lymphoma (GVL) effect. In theory, withdrawal of the immunosuppressive agents typically given following HSCT or use of nonmyeloablative preparation regimens in those with minimal disease prior to transplantation would allow engraftment and cause the host’s antigen-presenting cells to present antigens to donor T cells, which then produce a GVL response [99,100]. Bishop et al. [101] demonstrated, in patients with DLBLCL that had relapsed or were not in CR by day 100 after HSCT, that withdrawal of immunosuppression or infusion of donor lymphocytes led to at least partial remission in 60% of patients, with patients remaining alive for at least 42 months. More than half of patients with a response also developed graft-versus-host disease (GVHD). In another recent study by Jeon et al. [102], patients with various forms of NHL who experienced chronic GVHD of greater than mild severity had better OS rates than did those with acute GVHD, indicating that chronic GVHD may lead to a GVL effect. These results demonstrate that a GVL effect can be induced to target NHL tumors following allogeneic HSCT. Reduced-intensity conditioning regimens administered before allogeneic HSCT have also been used to induce a GVL effect. In a mixed leukemia–lymphoma study of over 1000 adult patients, Storb et al. [103] demonstrated that patients with any stage of NHL who received reduced-intensity conditioning with total body irradiation with or without fludarabine had the lowest risk of relapse among all comparison groups. These data indicate that reduced-intensity conditioning not only reduces side effects associated with preparative regimens prior to HSCT but also allows engraftment that can sustain the GVL effect.

## 5. Advances in the Treatment of Pediatric and Adolescent NHL

While conventional chemotherapy has positive results in pediatric and adolescent patients with NHL, especially in those with Burkitt lymphoma or DLBCL, the outcomes of patients with R/R NHL are dismal, even when HSCT is used. Moreover, chemotherapy, including preconditioning chemotherapy prior to HSCT, carries serious adverse effects such as mucositis, decreased appetite, nausea and vomiting, and susceptibility to infections, which can hinder patients’ recovery [104]. Discussed below are promising new drugs and immunotherapies for the treatment of NHL including R/R NHL. We also discuss areas where there is a need for further research into the application of these treatment modalities for pediatric or adolescent patients with NHL. These modalities are further summarized in Table 2.

### 5.1. Targeting Cell-Surface Molecules and DNA Replication

Rituximab is a monoclonal antibody that binds the CD20 receptors on lymphoma cells, leading to antibody-dependent and complement-mediated cytotoxicity. It has produced favorable results in studies of adults with high-risk lymphomas [143]. In a 2020 study, Minard-Colin et al. [77] analyzed the effects of adding rituximab to the LMB chemotherapy backbone, mainly for patients with Burkitt lymphoma and DLBCL. Pediatric patients with high-risk disease who received rituximab plus LMB had a significantly higher (one sided *p*-value of 0.00096) EFS rate than did similar patients who received LMB alone (93.9% vs. 82.3%, respectively). In addition, a COG pilot study showed that rituximab is safe when combined with standard chemotherapy and resulted in 3-year EFS rates of 95% for patients with intermediate-risk FAB group B (stage III/IV) disease and 90% for those with high-risk FAB group C disease [104,144]. Outcomes were comparable for patients with and without CNS involvement [104,145,146]. Moreover, the use of high-dose methotrexate for early CNS prophylaxis with an alternating regimen of R-CHOP and R-CHOP plus etoposide has not only shown improved failure-free-survival (FFS) rates of 74%, but a CNS progression rate of 2.3%, a decrease from 5% in regimens involving R-CHOP only [147]. Most recently, the European Intergroup/COG Inter-B-NHL Ritux 2010 trial, which randomized high-risk pediatric patients with Burkitt lymphoma or DLBCL to chemotherapy with or without rituximab, was terminated early because it found a substantial benefit in the rituximab arm, with a 1-year EFS rate of 94.2% compared to 81.5% in the control arm [77,104]. Further, in a study of pediatric patients with advanced (stage III/IV) NHL, Goldman et al. [148] demonstrated that the use of rituximab was associated with a 60% reduction in the anthracycline dose needed to maintain acceptable EFS and OS rates. Indeed, the researchers showed that rituximab may permit reduction of anthracycline dosing to 50 mg/m^2^ total, in light of the 100% EFS and OS rates at 4 years in the FAB group B arm. Taken together, these studies indicate that rituximab has great utility for treating CD20-positive NHL, including advanced-stage disease, and can permit reduction of conventional chemotherapy dosages to reduce adverse effects. In December 2021, the FDA approved rituximab for use in combination with chemotherapy in patients between the ages of 6 months and 18 years with DLBCL or Burkitt lymphoma on the basis of the results of the Inter-B-NHL-Ritux 2010 trial [77,149].

Brentuximab vedotin (BV) is a CD30-directed antibody conjugated to the microtubule-disrupting agent monomethyl auristatin E and works to induce apoptosis in CD30-positive cells during mitosis [150]. Because it targets receptors on lymphoma cells, BV has the added benefit of fewer off-target toxic effects than systemic chemotherapy [151]. In a phase 1/2 study of BV in pediatric patients with classical Hodgkin lymphoma and ALCL, 77% of patients had a reduction in tumor size, and the OS rate at 12 months was 79%; moreover, the safety profile was no worse than that observed in adults [151]. In the COG trial ANHL12P1, which included pediatric patients with ALCL, the addition of BV to conventional chemotherapy led to no additional toxic effects, including no grade 3 or 4 neuropathy and 2-year OS was 97% and 2-year EFS was 79%, indicating that BV may be useful when added to chemotherapy regimens for pediatric patients with ALCL [109]. BV also shows promise as an adjunct agent to help induce remission in patients with ALCL and as a bridging immunotherapy while patients await HSCT.

Blinatumomab is a bispecific T-cell-engaging antibody that targets the CD19 receptor on leukemia cells. When used in the postinduction phase for treatment of relapsed B-cell ALL in pediatric patients, it has been shown to improve EFS and OS rates [152,153]. Blinatumomab links the targeting regions of two antibodies directed against CD19 and CD3. CD19 is expressed by precursor-B-ALL cells, and CD3 is a constant part of the T-cell receptor complex [152,153]. While blinatumomab has been associated with acute side effects such as cytokine release syndrome (CRS) and neurotoxicity, there do not appear to be significant long-term neurotoxic effects in adult patients with R/R NHL treated with blinatumomab [154]. While the use of blinatumomab has not been approved for pediatric patients with NHL, phase 1 and 2 trials have shown that blinatumomab has great promise. Goebler et al. [155,156] demonstrated overall response rates (ORRs) of nearly 70% for patients with all NHLs and 55% for patients with DLBCL with a dose of 60 µg/m^2^ per day serving as the maximum tolerated dose. Dufner et al. [154] found similar results; in their study, use of blinatumomab at a maximum tolerated dose of 60 or more µg/m^2^ per day led to a median OS duration of nearly 8 years in patients with R/R NHL, with minimal to no long-term adverse effects. In contrast, patients treated at a dose below 60 µg/m^2^ per day had a median OS of just over 1 year. Considering that early phase trials have shown that blinatumomab has some activity in adults with R/R NHL, further prospective and retrospective studies are needed to determine the maximum tolerated dose in the pediatric NHL population and the impact of blinatumomab treatment on EFS and OS, especially in patients with R/R NHL [154,157].

Another cell surface effector, Inotuzumab ozogamicin is a CD22-directed antibody that is conjugated with calicheamicin, a cytotoxin that leads to the death of CD22-expressing leukemia cells. In several clinical trials, inotuzumab ozogamicin has been shown to induce remission of R/R ALL with minimal off-target effects owing to its specificity for the CD22 receptor on leukemia cells [158]. While phase 2 studies of inotuzumab ozogamicin are currently ongoing for adults with R/R NHL, including those whose tumors are not responsive to rituximab, no data has been published for the use of inotuzumab in pediatric and adolescent population with NHL. Thus, this is an area in which further research focused on pediatric NHL is required especially considering that the role of inotuzumab in the treatment of R/R pediatric ALL has been positive in ongoing early phase trials from the Children’s Oncology Group (Clinicaltrials.gov identifier NCT02981628) and a European-based multi-institutional study, ITCC-059, which shows an overall response rate of over 80% in patients with R/R CD22+ ALL who were treated with inotuzumab. Considering that these studies are enrolling patients with B-cell lymphoid malignancies, including NHL, there is a possibility that inotuzumab may be associated with positive outcomes in the treatment of CD22+ R/R NHL [104,120,159]. However, it must be noted that inotuzumab has been associated with increased risk of veno-occlusive disease (VOD) in those patients subsequently receiving an allogenic transplant, which can be fatal [160].

Glofitamab and mosunetuzumab have also emerged as bi-specific antibodies showing promise in the treatment of R/R NHL [161]. Although currently in early phase clinical trials, glofitamab, a bi-specific antibody that binds the CD3 receptors on T cells and CD20 receptors on B cells, has been studied in combination with R-CHOP with great response. A phase IB clinical trial has shown that the combination regimen induces an OR of nearly 90% at 9 months in patients with R/R NHL and a CR of 100% in patients in patients with untreated DLBCL [162]. Moreover, CRS rates were found to be significantly low with 3% of patients experiencing ≥ grade 3 CRS in the R/R NHL group and no patients experiencing CRS in the upfront DLBCL group. Mosunetuzumab, a similar CD3-CD20 bi-specific engager, when used as a single agent has shown an objective response rate of 35% in those with aggressive, R/R NHL and an objective response rate of 66% in those with indolent, R/R NHL [163]. Similar to glofitamab, the CRS rate of those experiencing ≥ grade 3 CRS was low with a rate slightly greater than 1%. Considering that these results are from a phase I trial, more data is needed to determine the role of mosunetuzumab as a single agent or in combination with conventional chemotherapy in those with initial or R/R disease.

Nelarabine is a purine analog and a form of deoxyguanosine that inhibits DNA synthesis in T lymphoblasts [164]. Initially approved in 2005 for the treatment of R/R T-cell lymphoblastic lymphoma in pediatric and adult patients, the drug has been shown to be tolerated well by pediatric patients. However, despite earlier reports indicating the utility of nelarabine monotherapy for R/R T-cell lymphoblastic lymphoma with response rates of nearly 40%, the COG AALL0434 study did not find significant differences in outcomes when nelarabine was added as an adjunct to the standard chemotherapy regimen [165,166,167,168]. These data are intriguing because nelarabine has shown an OR rate as high as 97% in studies of adults with T-cell lymphoblastic lymphoma when used with combination chemotherapy. Thus, further research into the optimization of nelarabine use in the pediatric and adolescent population is required [169].

### 5.2. Cellular Therapy

CAR T-cell therapy has produced good results in pediatric R/R leukemia but has not been extensively studied in the pediatric NHL population [170]. CAR T-cell therapy relies on the modification of a patient’s T lymphocytes in order to express a cell surface receptor, most commonly for CD19, that can recognize leukemia cells expressing the CD19 surface antigen and thereby lead to on-target cytotoxicity with minimal off-target effects [171]. While four different anti-CD19 CAR T-cell products have been approved for use in adults, none have been approved yet for pediatric patients with NHL [172]. Various CAR T-cell products such as tisagenlecleucel, KTE-X19, and axicabtagene clioleucel have been tested in phase 2 and 3 trials in adult patients with R/R LBCL and have shown ORRs ranging from 50 to 80% and complete response rates (CRRs) of 40–50%, in some cases indicating improvements in response over other salvage treatment options [173,174,175,176]. These same studies also demonstrated that the most common adverse effects such as anemia and neutropenia were similar to those seen in standard of care salvage therapies and grade 3 CRS and neurotoxicity, known adverse effects of CAR T-cell therapy, were often manageable with little to no associated mortality. Curiously, the BELINDA phase 3 trial is the most recent study on the use of tisagenlecleucel in patients with high-risk R/R LBCL which indicates that it does not have better outcomes than standard salvage therapy and the CAR T-cell product was not superior to the standard product [177]. In the upfront setting, the ZUMA-12 study, a phase 2 trial in adult patients with high-risk LBCL who received axicabtagene cliolecuel following 2 cycles of either R-CHOP or DA-EPOCH-R, has demonstrated that axicabtagene clioleucel may have an effective role as a first-line therapy considering that patients demonstrated an ORR greater than 80% with tolerable CRS and neurotoxicity [178].

Zhang et al. [179] have also published promising preliminary results of a clinical trial in which pediatric patients with Burkitt lymphoma, DLBCL, and B-cell lymphoblastic lymphoma underwent multiple rounds of anti-CD19, anti-CD20, or anti-CD22 CAR T-cell therapy, with an ORR of 94% and a CRR of 71%. Of note, 53% of patients experienced severe cytokine release syndrome, and 41% experienced some form of neurotoxicity including seizures. At this time, the BIANCA trial (NCT0310724), a clinical trial to determine the safety and efficacy of tisagenlecleucel, an anti-CD19 CAR T-cell therapy, is underway and includes pediatric, adolescent, and young adult patients with R/R B-cell NHL such as Burkitt lymphoma, DLBCL, and PMLBCL. Thus far, adverse effects have been tolerable, with no patients having cytokine release syndrome greater than grade 3 in severity [180]. CAR T-cell therapy is thus a potential treatment modality for patients with R/R NHL on the basis of its promising preliminary results in pediatric patients and its positive outcomes in adult patients with NHL [181].

While no large trials have yet evaluated the use of axicabtagene ciloleucel in pediatric patients with NHL, a case report of a 19-year-old with PMLBCL receiving axicabtagene ciloleucel indicated positive results with manageable adverse effects such as immune-effector cell-associated neurotoxicity syndrome (ICANS) [182]. These results indicate that axicabtagene ciloleucel is not only effective in inducing a response in R/R large B-cell lymphoma, but the response is sustained and possibly superior to the standard of care regimen of salvage chemotherapy followed, in those patients who respond, by high dose chemotherapy followed by stem cell rescue in patients with R/R disease. Taking into account the positive results of the ZUMA-7 trial, axicabtagene ciloleucel for the treatment of R/R LBCL was approved by the FDA in April 2022, joining tisagenlecleucel and lisocabtagene maraleucel as other anti-CD19 CAR T-cell products approved by the FDA for the treatment of R/R LBCL 177, [183,184,185]. The latter was approved in February 2021 after the phase 1 TRANSCEND NHL 001 trial indicated an ORR of over 70% and a CRR over 50%, with grade 3 or 4 CRS occurring in 1% of patients and grade 3 or 4 neurotoxicity in 10% of patients [184].

The phase 1/2 ZUMA-4 trial is underway to evaluate the anti-CD19 CAR T-cell therapy, KTE-X19, in the treatment of patients aged 21 years and under with DLBCL, PMLBCL, and Burkitt lymphoma that is refractory to or has failed two lines of therapy [186]. Preliminary results of the phase 1 portion of ZUMA-4 in patients with ALL are promising, with complete remission rates ranging from 64% to 75% across the various dosing groups. Moreover, 100% of patients with responses had negative minimal residual disease [187]. Thus, the preliminary outcomes of these trials indicate a rationale for the expansion of CAR T-cell products for the treatment of R/R pediatric NHL.

In patients in whom anti-CD19 CAR T-cell therapy fails, targeting CD22 may be an alternative. A current clinical trial has demonstrated that in patients whose large B-cell lymphoma has relapsed after CD19 CAR T-cell therapy, infusion of CD22-directed CAR T cells is safe and can lead to a complete remission lasting as long as 8 months [188]. While the most common CAR T-cell products singly target the CD19 receptor on cancer cells, researchers have also engineered dual-targeting CAR T-cells that recognize both CD19 and CD22 receptors on lymphoma cells. A recent study by Hu et al. [189] evaluated the effect of dual CD19/CD22 CAR T-cell products on R/R B-cell lymphoma; over 60% of patients had a CR, with a 2-year OS rate of 77% and PFS rate of 40%. In contrast, a phase 1 trial found that the use of dual anti-CD19/CD22 CAR T-cell therapy led to a 6-month PFS rate similar to that of tisagenlecleucel (29%), leading to closure of the large B-cell lymphoma arm [190]. Considering that response rates are generally worse in adults with large B-cell lymphoma than in children and that these studies primarily enrolled adult patients, extrapolation to the pediatric and adolescent population may be difficult, indicating that further trials are needed to determine the response of dual-targeting CAR T cells against pediatric NHL. Compounds which improve CAR T-cell killing such as decitabine may also serve as supplemental treatments to ensure long-term killing of CAR T-cells in order to avoid exhaustion and subsequent relapse [191,192]. Indeed, multiple early phase clinical trials exist to determine the use of decitabine primed CAR T-cells in their role in treating R/R NHL (clinicaltrial.gov identifiers, NCT04697940, NCT04850560) [193].

Another form of cellular therapy involves the use of antigen-specific T-cells directed towards other targets on NHL cells. One such modality that has shown promising results is EBV-specific T-cells from an autologous source, which target the latent membrane protein on EBV-associated NHL cells [194,195]. A study by Bollard et al. [195] included pediatric and adolescent patients and showed that infusion of cytotoxic T lymphocytes that target the latent membrane proteins on EBV-associated NHL or Hodgkin lymphoma cells produced durable remissions in more than 80% of patients at 2 years following infusion. Moreover, no patients experienced infusion-related toxicities.

Natural killer (NK) cells can recognize and kill tumor cells with minimal toxicity, as demonstrated in studies where NK cells have been infused alongside or in close timing with HSCT and demonstrated expansion and antileukemia effects, especially in acute myeloid leukemia [196]. While no large trials exclusively in pediatric and adolescent patients have determined the utility of NK cell infusion for the treatment of NHL, Liu et al. [197] have demonstrated in early-phase trials that NK cells engineered with an anti-CD19 CAR induced responses in four of six patients with R/R NHL at a median follow-up time of nearly 14 months. In addition, the NK cells persisted for at least 12 months following infusion. Moreover, a phase 2 clinical trial of patients older than 15 years with R/R NHL is currently studying the safety and 30-day mortality risk of a combination of NK cells derived from umbilical cord blood along with rituximab, chemotherapy, and HSCT; the study will also determine EFS, OS, and the persistence of the cord blood-derived NK cells [198]. Thus, NK cells are an exciting modality for the treatment of R/R NHL with minimal off-site toxicities.

Donor lymphocyte infusion (DLI) is another example of a modality that can induce a GVL effect in patients with NHL. In a 2018 retrospective registry analysis by Robinson et al. [199], adult patients with NHL who experienced relapse after allogeneic HSCT and then received DLI had a median response duration of 36 months; patients with follicular lymphoma had the highest OR rate among the NHL subtypes, 84%, with nearly 70% having CR [199]. While no large studies have yet determined the optimal use of DLI in pediatric patients with NHL, Gozdzik et al. [133] found that DLI in pediatric patients following HSCT, including those with Hodgkin lymphoma and NHL, led to a CR rate of 35%, although response rates were not broken down by cancer type. Moreover, 4% of patients died of DLI-induced GVHD. There is an obvious need for further study of the role of DLI in the treatment of pediatric NHLs in order to determine its efficacy, safety, and short- and long-term complications, including GVHD.

Tumor-infiltrating lymphocytes (TILs) are similar to NK cells in that they are an autologous source of cellular therapy that can be engineered to target certain tumor cells [200]. Researchers have demonstrated that administration of TILs with cyclophosphamide for the treatment of metastatic melanoma has a response rate of nearly 50%, with complete responses lasting more than 20 months and partial responses lasting a median of 4 months [201]. While studies have not determined the response of NHL to TIL therapy, researchers have shown that TILs can be isolated and engineered from lymphomatous tumors such DLBCL and follicular lymphoma, indicating that they may have future utility in the treatment of NHL [202].

### 5.3. Immune Checkpoint Inhibitors

In June 2018, the FDA approved the use of pembrolizumab for the treatment of adult and pediatric R/R PMLBCL [203]. Pembrolizumab is a small-molecule inhibitor targeting programmed cell death protein-1 (PD-1) on lymphocytes. This prevents tumor-secreted programmed cell death protein ligand-1 (PD-L1) from attaching and leading to lymphocyte deactivation and tumor evasion of immune system detection [204]. In the KEYNOTE trial, patients with R/R PMLBCL who received pembrolizumab tolerated the drug well, with none experiencing grade 5 adverse events and nearly 50% showing an overall response [205]. Current trials are also underway in testing the concurrent use of pembrolizumab with a host of other drugs, including rituximab and other immunotherapies such as CAR T-cell therapy, in order to improve outcomes in patients with R/R PMLBCL [203].

Phase 1/2 trials of another PD-1 inhibitor, nivolumab, showed safety and efficacy in pediatric patients with solid tumors and R/R classical Hodgkin lymphoma; however, its efficacy in the treatment of aggressive NHL remains unclear [137]. In the adult population, nivolumab has shown favorable tolerability, but the ORR is dismal in those with R/R disease, as evidenced by objective response rates of 10% in patients whose disease did not respond to autologous HSCT and 3% in patients who did not receive autologous HSCT [206]. Atezolizumab, a PD-L1 inhibitor, was also well tolerated in a large phase 1/2 study in pediatric patients with solid tumors, classical Hodgkin lymphoma, and NHL but its responses were also suboptimal, as only 5% of patients had a partial response at 6 months [207].

### 5.4. Targeted Therapy

As discussed above, nearly 90% of patients with ALCL harbor the ALK-NPM fusion protein on tumor cells, which can serve as a target for immunotherapy [208]. One such agent is crizotinib, a tyrosine kinase inhibitor that targets the ALK-ROS1-MET pathway in non-small cell lung cancer [209]. In a COG study, crizotinib monotherapy showed response rates of 80–90%, indicating that for patients with unresectable ALCL, crizotinib may be an acceptable form of immunotherapy and a bridge to HSCT [210]. On the basis of results from the COG ADVL0912 trial, in which nearly 40% of patients had a response lasting for up to 6 months, in January 2021 the FDA approved crizotinib for use in pediatric patients with R/R ALCL [211].

JAK2 inhibition may be useful in PMLBCL, which has been found to have mutations in pathways related to JAK/STAT signaling. In preclinical mouse xenograft models, JAK2 inhibition led to decreased tumor growth and longer survival in models of MLBCL and Hodgkin lymphoma [212]. In contrast, a small-scale study of patients greater than 19 years old detailed the effectiveness of Ruxolitinib, a JAK/STAT inhibitor, in the treatment of Hodgkin lymphoma but there was no response in PMLBCL. These studies underscore the need for larger trials that study the use of Ruxolitinib in the treatment of pediatric and adolescent PMBLCL or other NHL that may show amplification of the JAK pathway [213].

## 6. Conclusions

NHL in pediatric and adolescent patients is distinct type from the NHLs seen in adults. While the outcomes of pediatric and adolescent patients with primary NHLs such Burkitt lymphoma and DLBCL are outstanding, survival rates markedly decrease following relapse [89]. HSCT provides some hope for patients with R/R disease, although the results depend on the type of pediatric NHL. Here, we highlighted areas of recent progress in the treatment of NHL. We emphasize that recent advances are characterized by targeting treatment to cell-surface markers and cell receptors deployed by the tumor to evade detection by the immune system. While the current evolution in treatment is promising, further research is needed in areas such as the use of CAR T-cell therapy and monoclonal antibodies, especially as these treatments have had promising safety and response profiles in other pediatric cancers such as leukemia.

## Figures and Tables

**Table 1:** The molecular biology, genetics, and most common treatment regimens for common NHLs in the pediatric and adolescent population are described.

NHL Subtype	Molecular Markers and Genetic Abnormalities	Treatment Regimens
Burkitt lymphoma [12,62,63,64]	CD19, CD20, immunoglobulin kappa or gamma light chains, IgM, CD10/CALLA *MYC* translocations *TP53* mutation t(8:14)(q24: q32), t(8;22)(q24;q11)	FAB/LMB Regimen: COG-C5961 (FAB/LMB-96): intravenous (IV) formulations of cyclophosphamide (CPM), vincristine, prednisone/prednisolone, doxorubicin along with intrathecal (IT) methotrexate (MTX); IV Ifosfamide, etoposide, and MTX in higher risk group only (Group C) Inter-B-NHL Ritux 2010: Addition of rituximab to the FAB/LMB backbone BFM Regimen: Involve IV formulation of Doxorubicin and CPM with doses lower than FAB/LMB; addition of ifosfamide, etoposide, and IV methotrexate including in lower and intermediate risk groups
Diffuse large B-cell lymphoma [62,63,64]	Germinal center B-cell type: CD10 and *BCL6* Activated B-cell type: MUM1 *MYC* locus translocations involving chromosome 8q24	Regimens similar to that used in BL FAB: COG-C5961 (FAB/LMB-96) Inter-B-NHL Ritux 2010 BFM Regimen
Primary mediastinal large B-cell lymphoma [64,65]	CD19, CD20, CD30, CD79 *JAK2, STAT, PDL1/2* mutations	(DA-EPOCH-R): etoposide, doxorubicin, cyclophosphamide, vincristine, rituximab Dismal response to FAB/LMB in comparison to responses seen in DLBCL (EFS of 66%) No protocolized regimen
Anaplastic large cell lymphoma [62,66,67,68]	CD30, t(2;5) translocations encoding NPM-ALK	NHL-BFM studies: PO dexamethasone, IV methotrexate, IV etoposide, IV doxorubicin, IV vincristine, IV Vindesine and IT triple chemotherapy with methotrexate, cytarabine, and prednisone APO Regimen: IV doxorubicin, PO prednisone, IV vincristine, PO 6-mercaptopurine and IT/IV methotrexate
Lymphoblastic lymphoma [45,69,70,71,72,73,74]	B-cell type: CD19, cytoplasmic CD79a, cytoplasmic CD22 CD10, CD24, PAX5, CD20 and CD34 expression is variable T Cell type: CD3, CD1a, CD2, CD4, CD5, CD7, CD8, CD10, CD44	COG AALL0434 (T-LL regimen): Augmented BFM regimen IV Vincristine, IV Doxorubicin, IV Daunorubicin, IT MTX, IT Cytarabine, PO prednisone, IM peg-asparaginase, PO dexamethasone, IV/IM Cytarabine, Capizzi-style IV MTX (C-MTX), PO 6-mercaptopurine, PO thioguanine C-MTX: escalating doses of IV MTX with initial dose of 100 mg/m2/day; no leucovorin rescue NHL-BFM-95 (including B-type LL) COG-A5971 (including B type LL): similar to COG AALL0434, except does not include C-MTX; use high dose MTX
Pediatric follicular lymphoma [52,62,75]	Few cases with IRF4/MUM1 expression; No widespread genetic abnormalities have been identified	NHL-BFM studies: 2–4 cycles of IV Doxorubicin, CPM, ifosfamide Surgery in localized cases R-CHOP

Abbreviations: Ara-C Cytarabine; BFM: Berlin–Frankfurt–Münster; CHOP: Cyclophosphamide–Doxorubicin–Vincristine–Prednisone; COG: Children’s Oncology Group; C-MTX: Capizzi-style IV Methotexate; CPM: Cyclophosphamide; DA-EPOCH-R: Etoposide–Prednisone–Vincristine–Cyclophosphamide–Doxorubicin–Rituximab; Dex: Dexamthasone; DNR: Daunorubicin; DXR: Doxorubicin; IE: Ifosfamide–Etoposide; IFO: Ifosfamide; IRF4: Interferon regulatory factor 4; IT: Intrathecal; IV: Intravenous; MTX: Methotrexate; PEG-ASP: PEG-Asparaginase; PO: oral; R-CHOP: Rituximab–Cyclophosphamide–Doxorubicin–Vincristine–Prednisone/prednisolone; TIT: Triple intrathecal therapy (MTX, Ara-C and Hydrocortisoine); VCR: Vincristine; VP-16: Etoposide; 6-MP: 6-mercaptopurine; 6-TG: Thioguanine.

**Table 2:** Recent advancements, including clinical trials and Food and Drug Administration (FDA) approved cellular and immunotherapies, in the treatment of pediatric and adolescent non-Hodgkin lymphoma are summarized below.

Targeting Cell-Surface Molecules and DNA Replication				
Agent	Class	Target	FDA Approval	Key Clinical Trials in Pediatrics
Rituximab	mAb	CD 20	Approved for CD20+ DLBCL, BL, or mature B-ALL in pediatrics [105]	NCT01516580; Inter-B-NHL Ritux 2010—phase 3 [106] NCT00057811; ANHL01P1—phase 2 [107]
Brentuximab vedotin (BV)	ADC conjugated to MMAE	CD30	Approved for cHL and ALCL in adults Not approved in pediatrics [108]	NCT01979536; ANHL12P1—phase 2 [109] NCT02729961—phase 1/2 [110] NCT02581631; CheckMate 436—phase 1/2 [111] NCT03703050; NIVO-ALCL—phase 2 [112]
Blinatumomab	BiTE	CD19-CD3	Approved for MRD + B-ALL after first or second remission and R/R B-ALL in adults and children [113]	NCT00274742—phase 1 [114] NCT01741792—phase 2 [115] NCT03605589; Pembro-EB-1701—phase 1 [116] NCT03117751; TOT17—phase 2/3 [117]
Inotuzumab ozogamicin	ADC	CD22	Approved for R/R B-ALL in adults Not approved in pediatrics [118]	NCT02981628; AALL1621—phase 2 [119]; ITCC-059—phase 2 [120]
Nelarabine	Purine analog	DNA cell synthesis	Approved for R/R T-ALL and T-LL in adults and pediatrics [121]	NCT00408005; AALL0434—phase 3 [122]
Cellular Therapy				
Tisagenlecleucel	CAR T-cell	CD19	Approved for R/R B-ALL in pediatrics R/R Large B Cell lymphoma in adults [123]	NCT03610724; BIANCA—phase 2 [124]
Axicabtagene Ciloleucel	CAR T-cell	CD19	Approved for R/R Large B Cell lymphoma in adults Not approved in pediatrics [125]	NCT02625480; ZUMA-4—phase 1/2 [126]
CD22 CAR T-cells	CAR T-cell	CD22	Not approved	NCT02315612—phase 1 [127] NCT04088864—phase 1 [128]
CD19/CD22 CAR T-cells	CAR T-cell	CD19/CD22	Not approved	NCT03448393—phase 1 [129]
LMP1/2 CTLs (ALCI—Group A)	Antigen-specific T cells	(LMP) on EBV-associated NHL	Not approved	NCT00062868; ALASCAR—phase 1 [130]
CAR-NK-CD19	NK cells	CD19	Not approved	NCT04796675; CAR-NK-CD19 cells—phase 1 [131]
Cord blood derived NK Cells	NK cells		Not approved	NCT03019640—phase 2 [132]
Donor lymphocyte infusion (DLI)	DLI	GVL	Not approved	Study by PPGHSCT [133]
Tumor-infiltrating Lymphocytes (TILs)	TILs	Tumor specific	Not approved	None
Immune checkpoint inhibitors				
Pembrolizumab	PD-1 inhibitor	PD-1	Approved for R/R cHL in adults and pediatrics Approved for R/R PMBCL in adults and pediatrics [134]	NCT02332668; MK-3475-051/KEYNOTE-051—phase 1/2 [135]
Nivolumab	PD-1 inhibitor	PD-1	Approved for R/R cHL in adults Not approved in pediatrics [136]	NCT02304458; ADVL1412—phase 1/2 [137] NCT02927769; CheckMate 744—phase 2 [138]
Atezolizumab	PDL-1 inhibitor	PDL-1	Not approved for adult or pediatric lymphoma [139]	NCT02541604—phase 1/2 [140]
Targeted therapy				
Crizotinib	TKI	ALK-ROS1-MET pathway	ALK-positive ALCL in pediatrics and young adults [141]	NCT00939770; ADVL0912—phase 1/2 [142]

Abbreviations: ADC: Antibody Drug Conjugate; ALCL: Anaplastic Large Cell Lymphoma; ALK: Anaplastic Lymphoma Kinase; Axi-cel: Axicabtagene Ciloleucel; B-ALL; B-cell Acute Lymphoblastic Leukemia; BiTE: Bispecific T-Cell engager; BL: Burkitt Lymphoma; BLL: B Lymphoblastic Lymphoma; CAR T-cell: Chimeric Antigen Receptor T cell; CD: cluster of differentiation; cHL; Classical Hodgkin Lymphoma; CTL: Cytotoxic T Lymphocytes; DLBCL: Diffuse Large B-Cell Lymphoma; DLI: Donor Lymphocyte Infusion; EBV: Epstein Barr virus; GVL: Graft Versus Lymphoma; LMP: Latent Membrane Protein; mAb: monoclonal antibody; MMAE: Monomethyl auristatin E; MRD: Minimal residual Disease; NCT; National Clinical Trial; NK cells: Natural Killer Cells; PD-1: programmed cell death protein-1; PDL-1: programmed cell death protein ligand-1; PMBCL: Primary mediastinal large B-cell lymphoma; PPGHSCT: Polish Pediatric Group for Hematopoietic Stem Cell Transplantation; R/R: refractory/relapsed; T-ALL: T-cell Acute Lymphoblastic Leukemia; TILs: Tumor Infiltrating Lymphocytes; Tisa-cel: Tisagenlecleucel; TKI: Tyrosine Kinase Inhibitor; T-LL; T-cell Lymphoblastic Lymphoma.
